# Needs of family members of patients in a coronary care unit

**DOI:** 10.31744/einstein_journal/2022AO6258

**Published:** 2022-03-07

**Authors:** Alue Constantino Coelho, Camila Takáo Lopes, Juliana de Lima Lopes, Vinicius Batista Santos, Alba Lucia Bottura Leite de Barros

**Affiliations:** 1 Universidade Federal de São Paulo Escola Paulista de Enfermagem São Paulo SP Brazil Escola Paulista de Enfermagem, Universidade Federal de São Paulo, São Paulo, SP, Brazil.

**Keywords:** Family, Inpatients, Patient-centered care, Critical care, Nursing, Humanization of assistance, Acute coronary syndrome, Intensive care units

## Abstract

**Objective:**

To identify the need of family members of patients hospitalized in a coronary intensive care unit and their degree of satisfaction with the care provided.

**Methods:**

An observational and cross-sectional study including family members of patients hospitalized in the coronary intensive care unit for acute coronary syndrome in Killip I or II. After the second visit of the same family member to the patient in the unit, a 43-item inventory of needs and stressors of family members was applied. Family members assessed each need for its importance and satisfaction using a four-point Likert scale. The scores in each dimension of importance and satisfaction were compared using the Wilcoxon test, considering a value of p<0.05 as significant.

**Results:**

One hundred family members were interviewed. The most important needs were related to assurance and information. Family members had satisfaction scores corresponding to be very satisfied or totally satisfied, but with lower scores when compared to the needs scores (p<0.01).

**Conclusion:**

The most important needs of family members of patients hospitalized in the coronary intensive care unit were related to assurance and information. Multiprofessional interventions involving better communication of patient information to family members should be incorporated into the coronary intensive care unit.

## INTRODUCTION

Family members of patients hospitalized in intensive care units (ICU) frequently have negative emotional reactions, such as stress, anxiety, depression, fear, guilt, anger, insecurity, anguish, acute stress disorder, and post-traumatic stress disorder. In addition to these issues, impaired sleep quality leads to high levels of physical and emotional exhaustion.^(1–3)^

Some factors contributing to these reactions include the unfamiliar environment, the complex level of technology used in intensive care environments, the clinical severity of the patients, the lack of clarification of doubts, the impossibility to stay full time with their loved one, and the communication failure.^(1,2)^

Studies indicate that the main needs of family members of patients in the ICU are those related to assurance, information, and proximity.^(4,5)^ To reduce the occurrence of negative emotional symptoms of family members, meet their needs and increase their level of satisfaction with the care provide, a series of interventions that meet the principles of the Patient and Family Centered Care (PFCC) model may be carried out.^(6)^

From the PFCC perspective, care delivery must meet the wishes, needs, and preferences of patients and their families.^(5–7)^ In this context, identifying the needs of family members and assessing satisfaction with care delivery are indispensable for the implementation of PFCC principles.

In 1979, Nancy Molter carried out the first study to identify the perceived needs of family members of critically ill patients. Based on this study, a questionnaire was developed with 45 items, representing the importance attributed by family members to certain needs.^(8)^ In 1986, this tool was restructured and named Critical Care Family Needs Inventory (CCFNI). In 1991, a construct validation study of the CCFNI was carried out with 677 family members of patients in the ICU, in which the items were grouped in five dimensions: support, comfort, information, proximity, and assurance.^(9)^

Internationally, this instrument has undergone several types of validation and cross-cultural adaptation in different languages, and in the last 5 years it was translated and adapted into Spanish.^(10)^ In Brazil, the instrument was cross-culturally adapted and named *Inventário de Necessidades e Estressores de Familiares em Terapia Intensiva* (INEFTI).”^(11,12)^

Although the instrument has been applied in various types of ICU, there is a gap regarding the needs of family members of patients in the coronary ICU, because these patients are often not in critical condition, but they are admitted to this environment for surveillance and cardiovascular clinical stabilization. In this sense, identifying the needs of these family members and their level of satisfaction with care delivery may support the establishment of institutional routines, especially, nursing interventions aimed at relieving the discomfort and suffering of family members.

## OBJECTIVE

To identify the needs of family members of patients hospitalized in a coronary intensive care unit and their degree of satisfaction with the care provided.

## METHODS

### Design of the study

A cross-sectional, descriptive study reported based on the Strengthening the Reporting of Observational Studies in Epidemiology (STROBE) guidelines.^(13)^

#### Study setting

Data collection was carried out in the coronary ICU of a large public university hospital in the city of São Paulo (SP). The ICU had six beds for patients with acute coronary syndrome (ACS), with an average of 40 admissions per month. Family visits to the patients occurred once a day for 2 hours, with no restrictions as to the number of visits, however, two visitors at a time were allowed. The family members waited at the door of the ICU, where there were two armchairs and a sofa with capacity for three seats. The patient's status was transmitted by the on-duty physician to the family member identified by the patient as responsible.

### Sample

The sample consisted of family members over 18 years of age of patients admitted to the coronary ICU for ACS without clinical signs of acute ventricular dysfunction, able to understand and respond to the instruments and who had already visited the patient in the hospital at least once.

Family members of patients in Killip III or IV were excluded, since the greater severity of these patients could directly influence the needs of family members. In this study, the term “family member” is defined as a member of the patient's family, living in the same household as the patient, who received information about the patient during visiting hours or whom the patient identified as significant in their care.

### Data collection

Data were collected from March 2016 to January 2018. At the time of the visit, family members who were visiting the patients for the second time were identified, and the objective of the study was presented. After accepting to participate in the research and signing the Informed Consent Form, the individuals were referred to a private room to answer the instruments in an interviewing.

Two instruments were applied. The first instrument referred to the sociodemographic characterization of the relatives and patients (age, gender, degree of kinship, marital status, religion, education, and family income). The second instrument was the INEFTI and aimed at assessing the importance family members attributed to a list of 43 needs, using a 4-point Likert-type scale (1=not at all important; 2=not very important; 3=very important; 4=absolutely important).^(11)^

In addition to attributing importance considering a list of needs, family members attributed the degree of satisfaction with the care provided, taking into account each need, also by means of 4-point Likert-type scale (1=not at all satisfied; 2=not very satisfied; 3=very satisfied; 4=totally satisfied).^(11)^

The list of 43 needs evaluated according to the degree of importance and satisfaction was grouped in five dimensions: support, which reflects the need of the family for resources and specialized help; information, which comprises the need of the family to understand the patient's condition; comfort, which relates to the wish of the family to be physically comfortable to reduce suffering and anguish; closeness, which refers to the need of the family to maintain their relationship with the patient; and assurance, related to the wish of the family to maintain or redefine hope about the patient's outcome.^(11,12)^

The INEFTI presented a reliability by Cronbach's alpha test of 0.79 in the total scale, regarding importance, and of 0.86, regarding satisfaction.^(14)^

### Data analyses

Data were analyzed in IBM SPSS, version 22.0. The absolute and relative frequency distributions, means and standard deviations were used to summarize the demographic characteristics of the participants. To analyze the needs of family members in relation to importance and satisfaction, the mean and standard deviation were calculated for each indicator and each dimension of the INEFTI. The frequency of items with score greater than or equal to three was calculated, considering that this score is related to higher importance and satisfaction.^(14)^ The Wilcoxon test was used to compare the differences between the importance and satisfaction of each dimension assessed by family members. The level of statistical significance was set at 0.05.

### Ethical aspects

The Ethics Committee of the *Escola Paulista de Enfermagem* of the *Universidade Federal de São Paulo* (UNIFESP) approved this study, # 1.327.637, CAAE: 50468915.8.0000.5505. This is in accordance with the guidelines governing research involving human beings. Participants information were maintained confidential, anonymous, and they had possibility of leaving the study at any time.

## RESULTS

A total of 460 family members were assessed for eligibility after their first visit to the patient. Among these, 360 were excluded: 100 patients were hospitalized for less than 24 hours, 98 patients were sedated or hemodynamically unstable, and 162 did not return to visit the patient 24 hours after the admission.

A final sample of 100 family members was obtained. Most family members were women, aged between 18 and 30 years, married, catholic, had a high school education and monthly income between one and three Brazilian minimum wages (Table 1).

**Table 1:** Sociodemographic characterization of family members of patients hospitalized in the coronary intensive care unit

Sociodemographic variables		n (%)
Gender		
	Women	71 (71)
	Male	29 (29)
Age range, years		
	18-30	28 (28)
	31-40	24 (24)
	41-50	17 (17)
	51-60	22 (22)
	61-70	7 (7)
	>70	2 (2)
Relationship with patient		
	Son/Daughter	53 (53)
	Partner	36 (36)
	Others	5 (5)
	Brother	4 (4)
	Boyfriend	1 (1)
	Mother	1 (1)
Marital status		
	Married	46 (46)
	Single	32 (32)
	Stable union	15 (15)
	Divorced	6 (6)
	Widow and Widowed	1 (1)
Religion		
	Catholic	47 (47)
	Evangelical	24 (24)
	Spiritualist	10 (10)
	Christian	9 (9)
	None	6 (6)
	Other	4 (4)

From the INEFTI list of 43 needs, 35 (81%) were considered important or very important, highlighting: “to know the patient's chance of improvement,” to be assured that the best care possible is being give to the patient,” to know specific facts concerning the patient's progress,” to know why things were done for the patient,” and to know how the patient is being treated medically.” Of the 35 indicators rated as important or very important by family members, six were rated with dissatisfaction or low satisfaction, highlighting: “having a person who can provide information over the phone,” “to have someone to help with financial problems”, and “to know that other professionals can help me” (Table 2).

**Table 2:** Needs of family members of patients hospitalized in the coronary intensive care unit related to importance and satisfaction

		Importance		Satisfaction	
		Mean ± SD	Score ≥3 n (%)	Mean ± SD	Score ≥3 n (%)
Assurance Dimension					
	1. To Know the expected outcomes	3.79±0.43	99 (99)	3.43±0.71	91 (91)
	5. To have questions answered honestly	3.69±0.56	97 (97)	3.50±0.64	95 (95)
	14. To feel that there is hope for improvement of the patient	3.64±0.50	100 (100)	3.52±0.62	97 (97)
	17. To be assured that the best care possible is being given to the patient	3.74±0.44	100 (100)	3.50±0.68	95 (95)
	33. To have explanations given that are understandable	3.64±0.50	99 (99)	3.44±0.68	93 (93)
	40. To feel that the hospital personnel care about the patient	3.69±0.48	99 (99)	3.51±0.67	96 (96)
	41. To know specific facts concerning the patient's progress	3.73±0.46	99 (99)	3.46±0.74	91 (91)
Support Dimension					
	2. To have explanations of the environment before going into the critical care unit for the first time	3.63±0.52	98 (98)	3.33±0.84	86 (86)
	7. Talking about negative feelings about what has happened	2.99±0.99	76 (76)	3.05±0.94	74 (74)
	9. To have directions as to what to do at the bedside	3.52±0.64	94 (94)	3.11±0.93	76 (76)
	12. To have friends nearby for support	3.04±0.72	90 (90)	3.19±0.80	86 (86)
	18. To have a place to be alone while in the hospital	2.56±1.04	52 (52)	3.00±0.91	77 (77)
	22. To have someone to help with financial problems	3.02±0.90	71 (71)	2.85±1.11	60 (60)
	24. To have someone from the religion I belong to visit	2.48±1.13	49 (49)	2.81±1.06	61 (61)
	25. To talk about the possibility of the patient's death	3.42±0.80	90 (90)	3.18±0.93	82 (82)
	26. To be accompanied by a professional, friend or family member during the visit	3.33±0.75	91 (91)	3.35±0.70	93 (93)
	27. To have someone be concerned with your health	3.42±0.68	91 (91)	2.99±1.13	68 (68)
	30. To feel it's alright to demonstrate feelings and emotions	3.07±0.93	80 (80)	3.09±0.94	76 (76)
	31. Knowing what other professionals can help me	3.39±0.69	90 (90)	2.85±1.00	63 (63)
	35. To be informed about religious services	2.56±1.02	56 (56)	2.67±1.10	58 (58)
Information Dimension					
	3. To talk to the doctor every day	3.67±0.55	96 (96)	3.51±0.62	95 (95)
	4. To have a person who can give information over the telephone	3.42±0.84	89 (89)	2.23±1.16	39 (39)
	11. To know who can give the information I need	3.54±0.59	95 (95)	3.24±0.88	82 (82)
	13. To know why things were done for the patient	3.72±0.47	99 (99)	3.47±0.75	92 (92)
	15. To know about the types of staff members taking care of the patient	3.64±0.50	99 (99)	3.43±0.71	93 (93)
	16. To know how the patient is being treated medically	3.73±0.44	100 (100)	3.47±0.73	92 (92)
	19. To know exactly what is being done for the patient	3.70±0.46	100 (100)	3.36±0.67	93 (93)
	36. To help to care for the patient in the ICU	2.97±1.03	71 (71)	3.17±0.85	58 (58)
Proximity Dimension					
	6. To have visiting hours changed for special conditions	3.16±0.91	79 (79)	2.96±1.00	70 (70)
	10. To visit at any time	2.68±1.10	58 (58)	2.79±0.99	62 (62)
	29. To talk to the same nurse every day	2.99±0.88	73 (73)	3.24±0.80	85 (85)
	34. To have visiting hours start on time	3.58±0.57	96 (96)	3.39±0.81	87 (87)
	37. To be informed about possible transfers	3.70±0.48	99 (99)	3.31±0.86	88 (88)
	38. To be called at home about changes in the patient's condition	3.70±0.55	97 (97)	2.93±1.13	67 (67)
	39. To receive information about the patient at least once a day	3.53±0.68	95 (95)	3.12±0.87	79 (79)
	42. To see the patient frequently	3.49±0.73	90 (90)	3.31±0.80	87 (87)
	43. To have the waiting room near the patient	3.33±0.80	89 (89)	3.39±0.77	90 (90)
Comfort Dimension		3.18±0.57		3.10±0.59	
	8. To have good food available in the hospital	2.84±1.02	67 (67)	2.86±0.93	65 (65)
	20. To have comfortable furniture in the waiting room	3.02±0.92	73 (73)	3.27±0.77	88 (88)
	21. To feel accepted by the hospital staff	3.38±0.78	87 (87)	3.38±0.70	91 (91)
	23. To have a telephone near the waiting room	2.88±0.99	64 (64)	2.67±1.05	55 (55)
	28. To be assured it is alright to leave the hospital for a while	3.67±0.49	99 (99)	3.46±0.68	95 (95)
	32. To have a bathroom near the waiting room	3.33±0.69	89 (89)	2.96±0.98	74 (74)
Global score		3.37±0.37		3.25±0.53	

SD: standard deviation; ICU: intensive care unit.

The dimensions considered most important were assurance, information, and proximity. The dimensions of satisfaction with the lowest scores were comfort, proximity, and information. All dimensions of INEFTI presented higher scores of importance when compared with the satisfaction scores, except for the Support Dimension and the global score, which were not significantly different (Table 3).

**Table 3:** Comparison between level of importance and level of satisfaction of family members of patients hospitalized in the coronary intensive care unit

Dimension	Importance	Satisfaction	p value*
	Mean±SD	Mean±SD	
Assurance	3.70±0.36	3.40±0.48	<0.001
Support	3.13±0.49	3.38±0.64	0.669
Information	3.51±0.38	3.23±0.59	<0.001
Proximity	3.35±0.47	3.16±0.62	0.004
Comfort	3.18±0.57	3.10±0.59	<0.001
Global score	3.37±0.37	3.25±0.53	0.07

* Wilcoxon test.

SD: standard deviation.

## DISCUSSION

The needs of families of hospitalized patients are often disregarded due to the complexity of the intensive care environment by the technical and reductionist behavior of providers, and the lack of training in appropriate communication with family members.^(15,16)^ For this reason, one of the intensive care nurses’ responsibilities is to identify the specific needs of family members and implement appropriate and essential interventions.^(17)^ In this study, the importance assigned to the needs of family members of patients hospitalized in a coronary ICU and the satisfaction with care were assessed and compared.

The main characteristics of PFCC in the ICU are to provide care that respects and responds to the individual preferences, needs, and values of patients and families, particularly, in an attempt to ensure that the wishes of patients and families are the guide for all clinical decisions. The key element of PFCC in the ICU is the active and direct involvement of family members and patients in decisions and self-care, ensuring physical and emotional comfort, with adequate understanding of the clinical status.^(18)^

This survey found that needs considered of greatest importance by family members were related to safety and information, mainly regarding the patient's treatment, with clear and precise information about patients’ clinical evolution, as shown in findings of another study. This finding corroborates other studies carried out in intensive care settings but not specifically in a coronary ICU.^(19,20)^ Communication is an important need of family members of patients in the ICU^(20)^ and is part of the professional-patient-family relationship.

Studies^(4,21)^ addressing identification of perceived needs of family members of critically ill patients confirm the need for honest and frequent information by families about the status of hospitalized patients, recovery, evolution and prognosis, corroborating the findings of this survey. For this reason, information must be transmitted using accessible and appropriate language, to meet and respect families’ needs.^(22)^

Providers’ attitudes, such as educating patients and relatives, increase satisfaction levels, reduce anxiety and improve the relatives’ understanding,^(6)^ regardless of the patients’ outcomes.^(18,21,22)^ Even in situations of unfavorable clinical progression, communication and support received by the multiprofessional team are the greatest predictors of family satisfaction.^(18,21,22)^

When families are allowed to play a more participatory role, a decrease in the number of phone calls occurs, without disregarding the family's need for information or compromising satisfaction with care delivery.^(18)^

The quality of communication depends on the characteristics and needs of each health care organization, the training of providers, the collaboration of the team, and providers’ attitudes toward families.^(21)^ The mnemonic rule developed by the University of Washington, called VALUE, can help health care professionals communicate effectively with family members in order to reduce their stress and anxiety: to value what family members say; to acknowledge family members’ emotions; to listen; to understand who the patient is as a person by asking family members questions, and to elicit questions from family members.^(23)^

Another aspect identified in previous studies, which corroborates the findings of the present study regarding the need for family members’ communication, is that family members’ participation in interdisciplinary visits improves decision-making and communication with the team. As a consequence, this improves family members’ satisfaction. Family presence and participation are fundamental for implementation of humanized care in the ICU,^(3,24)^ however, at the setting where this survey was conducted, family members did not participate in the multiprofessional visits. This non-participation possibility may explain the lower satisfaction in the information and support dimensions compared with the scores of needs in the same domains.

In our study, all dimensions of satisfaction evaluated had mean scores greater than or equal to 3, thereby, demonstrating that the level of satisfaction of the families with the service was adequate. The Comfort Dimension had the lowest satisfaction scores, especially about the physical structure, such as the importance of having a bathroom close to the waiting room, telephone, and the welcoming of family members. This finding was also identified in another study conducted in Brazil,^(7)^ in which the lowest level of satisfaction was found in the Comfort Dimension.

Measures must be taken to improve the physical structure and welcoming of family members, including noise reduction in the ICU and individual rooms, with space reserved for family members, as described in the PFCC implementation guidelines.^(18)^

These results are limited because they were carried out in only one center, which may limit generalization of results. In addition, this study was comprised of a convenience sample, and the levels of satisfaction with the needs that were met may have been underestimated, since the responses to the INEFTI may have been affected by social desirability. In this sense, periodic reassessments are indicated, including the assessment of the impact of improvement interventions.

## CONCLUSION

The most important needs of family members of hospitalized patients in the coronary intensive care unit were related to safety and information - especially the communication established with members of the health care team. Family members reported being satisfied or very satisfied with the fulfillment of all dimensions of needs. Multiprofessional interventions involving better communication of information about patients to family members must be incorporated in the coronary intensive care unit.

## References

[1] 1 Hekmatpou D, Ebrahimi-Fakhar HR. Addressing disruption in family life: exploration of the perceived needs of the families of patients hospitalized in critical care units in Iran. J Nurs Res. 2015;23(2):118-24. 10.1097/JNR.0000000000000069 25967642

[2] 2 Vasconcelos EV, Freitas KO, Silva SE, Baia RS, Tavares RS, Araújo JS. The daily life of relatives of patients admitted in icu: a study with social representations. J Rev Fundam Care Online. 2016;8(2):4313-27.

[3] 3 Reis CC, Sena EL, Fernandes MH. Humanization care in intensive care units: integrative review. J Rev Fundam Care Online. 2016;8(2):4212-22.

[4] 4 Meneguin S, Matos TD, Miot HA, Pollo CF. Association between comfort and needs of ICU patients’ family members: a cross-sectional study. J Clin Nurs. 2019;28(3-4):538-44. 10.1111/jocn.14644 30091154

[5] 5 Goldfarb MJ, Bibas L, Bartlett V, Jones H, Khan N. Outcomes of patient- and family-centered care interventions in the ICU. a systematic review and meta-analysis. Crit Care Med. 2017;45(10):1751-61. Review. 10.1097/CCM.0000000000002624 28749855

[6] 6 The Joint Commission. Transitions of care: engaging patients and families. Quick Safety. 2015:18:1-3 [cited 2020 Dec 21]. Available from: https://www.jointcommission.org/assets/1/23/Quick_Safety_Issue_18_November_20151.PDF

[7] 7 Davidson JE, Zisook S. Implementing family-centered care through facilitated sensemaking. AACN Adv Crit Care. 2017;28(2):200-9. 10.4037/aacnacc2017102 28592480

[8] 8 Molter NC. Needs of relatives of critically ill patients: a descriptive study. Heart Lung. 1979;8(2):332-9. 253712

[9] 9 Leske JS. Internal psychometric properties of the Critical Care Family Needs Inventory. Heart Lung. 1991;20(3):236-44. 2032860

[10] 10 Rojas Silva N, Padilla Fortunatti C, Molina Muñoz Y, Amthauer Rojas M. The needs of the relatives in the adult intensive care unit: cultural adaptation and psychometric properties of the Chilean-Spanish version of the critical care family needs inventory. Intensive Crit Care Nurs. 2017;43:123-8. 10.1016/j.iccn.2017.07.006 28917604

[11] 11 Castro DS. Estresse e estressores dos familiares de pacientes com traumatismo crânio - encefálico em terapia intensiva [tese]. Rio de Janeiro (RJ): Escola de Enfermagem Anna Nery, Universidade Federal do Rio de Janeiro; 1999. 144 p.

[12] 12 Morgon FH, Guirardello EB. Validation of a ratio scale for family needs at an intensive care unit. Rev Lat Am Enfermagem. 2004;12(12):198-203. 10.1590/s0104-11692004000200008 15303223

[13] 13 Von Elm E, Altman DG, Egger M, Pocock SJ, Gotzche PC, Vandenbroucke JP. The Strengthening the Reporting of Observational Studies in Epidemiology (STROBE) statement: guidelines for reporting observational studies. J Clin Epidemiol. 2008;61(4):344-9. 10.1016/j.jclinepi.2007.11.008 18313558

[14] 14 Freitas KS, Kimura M, Ferreira KA. Family members’ needs at intensive care units: comparative analysis between a public and a private hospital. Rev Lat Am Enfermagem. 2007;15(1):84-92. 10.1590/s0104-11692007000100013 17375237

[15] 15 Grinberg AR, Tripodoro VA. Futilidad médica y obstinación familiar en terapia intensiva ¿Hasta cuándo seguir y cuándo parar? Medicina. 2017;77(6):491-6. 29223941

[16] 16 Pecanac K, King B. Nurse-family communication during and after family meetings in the intensive care unit. J Nurs Scholarsh. 2019;51(2):129-37. 10.1111/jnu.12459 30697910

[17] 17 Kynoch K, Chang A, Coyer F, McArdle A. The effectiveness of interventions to meet family needs of critically ill patients in an adult intensive care unit: a systematic review update. JBI Database Syst Rev Implement Reports. 2016;14(3):181-234. Review. 10.11124/JBISRIR-2016-2477 27532144

[18] 18 Society of Critical Care Medicine (SCCM). The intensive care professionals. SCCM family-centered care guidelines supplement: work tools for guideline implementation. Last Updated 03.08.2020. Los Angeles (CA): SCCM; 2020 [cited 2020 Dec 21]. Available from: https://www.sccm.org/getattachment/736d536f-4e97-4457-bdcc-365d69829e45/SCCM-Family-Centered-Care-Guidelines-Supplement-W

[19] 19 Batista VC, Monteschio LV, Godoy FJ, Goes HL, Marcon SS, Matsuda LM. Needs of the relatives of patients hospitalized in an intensive therapy unit. Rev Fund Care Online. 2019;11(2):540-6.

[20] 20 Khatri Chhetri I, Thulung B. Perception of nurses on needs of family members of patient admitted to critical care units of teaching hospital, Chitwan Nepal: a cross-sectional institutional based study. Nurs Res Pract. 2018;1369164. 10.1155/2018/1369164 PMC6038657 30046491

[21] 21 Velasco Bueno JM, Alonso-Ovies A, Heras La Calle G, Zaforteza Lallemand C; Equipo de investigación del Proyecto HUCI (Humanizando los cuidados intensivos). Main information requests of family members of patients in Intensive Care Units. Med Intensiva (Engl Ed). 2018;42(6):337-45. 10.1016/j.medin.2017.09.007 29108720

[22] 22 Quenot JP, Ecarnot F, Meunier-Beillard N, Dargent A, Large A, Andreu P, et al. What are the ethical issues in relation to the role of the family in intensive care? Ann Transl Med. 2017;(Suppl 4):S40. Review. 10.21037/atm.2017.04.44 PMC5750250 29302596

[23] 23 Lautrette A, Darmon M, Megarbane B, Joly LM, Chevret S, Adrie C, et al. A communication strategy and brochure for relatives of patients dying in the ICU. N Engl J Med. 2007;356(5):469-78. 10.1056/NEJMoa063446 17267907

[24] 24 Kang J, Cho YJ, Choi S. State anxiety, uncertainty in illness, and needs of family members of critically ill patients and their experiences with family-centered multidisciplinary rounds: a mixed model study. PLoS One. 2020;15(6):e0234296. 10.1371/journal.pone.0234296 PMC7282650 32516349

